# Association of CD166 expression with clinicopathologic characteristics of colorectal cancer

**Published:** 2016

**Authors:** Dariush Moslemi, Mohammad Mohammadianpanah

**Affiliations:** 1 **Division of Radiation Oncology, School of Medicine, Babol University of Medical Sciences, Babol, Iran.**; 2 **Colorectal Research Center, Shiraz University of Medical Sciences, Shiraz, IR Iran.**

**Keywords:** CD166 expression, Pathology, colorectal cancer, characteristics

In the fall 2013 issue of Caspian Journal of Internal Medicine, an interesting article entitled “The association between CD166 detection rate and clinicopathologic parameters of patients with colorectal cancer” was reported by Shafaei et al. (1). The authors investigated an association between CD166 expression and clinicopathologic characteristics of colorectal cancer. 

They found an association between cytoplasmic expression of CD166 and non-mucinous type of colorectal cancer. However, some important pathological data, including tumor size, total number of identified lymph nodes, number of positive lymph nodes, presence of lymphatic and perineural invasion, and presence of obstruction and/or perforation were missed out. These pathological characteristics might be important indicators for defining accurate tumor and lymph node staging and predicting the prognosis (2, 3). 

In tables 1 and 2, no p-value was provided for the tumor stage and grade; because some values are too small for a valid statistical analysis. Therefore, the authors should check the test assumptions of the tests and redo the analyses after combining categories when needed, for example, consider combining T1-2 against T3-4; as well G1 versus G2-3 ones. Furthermore, it is not clear why the authors classified primary sites to the left and right colon and included the rectum to the left colon. They should have reclassified the primary sites to the colon and the rectum; or as left colon, right colon and the rectum. 
